# Deciphering a survival strategy during the interspecific competition between *Bacillus cereus* MSM-S1 and *Pseudomonas* sp. MSM-M1

**DOI:** 10.1098/rsos.160438

**Published:** 2016-11-23

**Authors:** Brinta Chakraborty, Anish Mallick, Sumana Annagiri, Supratim Sengupta, Tapas K. Sengupta

**Affiliations:** 1 Department of Biological Sciences, Indian Institute of Science Education and Research (IISER) Kolkata, Mohanpur 741246, India; 2 Department of Mathematical Sciences, Indian Institute of Science Education and Research (IISER) Kolkata, Mohanpur 741246, India; 3 Department of Physical Sciences, Indian Institute of Science Education and Research (IISER) Kolkata, Mohanpur 741246, India

**Keywords:** *Pseudomonas*, *Bacillus*, interspecific interaction, mathematical model

## Abstract

Interspecific competition in bacteria governs colony growth dynamics and pattern formation. Here, we demonstrate an interesting phenomenon of interspecific competition between *Bacillus cereus* MSM-S1 and *Pseudomonas* sp*.* MSM-M1, where secretion of an inhibitor by *Pseudomonas* sp*.* is used as a strategy for survival. Although *B. cereus* grows faster than *Pseudomonas* sp., in the presence of *Pseudomonas* sp. the population of *B. cereus* reduces significantly, whereas *Pseudomonas* sp. do not show any marked alteration in their population growth. Appearance of a zone of inhibition between growing colonies of two species on nutrient agar prevents the expanding front of the MSM-S1 colony from accessing and depleting nutrients in the region occupied by MSM-M1, thereby aiding the survival of the slower growing MSM-M1 colonies. To support our experimental results, we present simulations, based on a chemotactic model of colony growth dynamics. We demonstrate that the chemical(s) secreted by *Pseudomonas* sp. is responsible for the observed inhibition of growth and spatial pattern of the *B. cereus* MSM-S1 colony. Our experimental results are in excellent agreement with the numerical results and confirm that secreted inhibitors enable *Pseudomonas* sp. to survive and coexist in the presence of faster growing *B. cereus*, in a common niche.

## 1. Introduction

Ecology is a scientific study pertaining to the relationship between living organisms and their environment. The coexistence of multiple species in an ecological niche with both intra- and interspecies interaction can help maintain the balance of the ecosystem. Previously, prokaryotes were thought to be solitary organisms that do not take an active part in maintaining ecological balance, but this idea has changed with the advancement of ecological and evolutionary studies [1,2]. With time, bacteria have evolved to cope with the changes in both biotic and abiotic environments. They have developed complex strategies [3–7] to thrive in hostile conditions that may be characterized by limited nutrients, the presence of toxic chemicals, competing species, predators and changes in temperature.

Individuals of similar and different species of bacteria can facilitate each other by employing cooperative strategies [8]. Different species of bacteria may also compete for resources, and competition for one resource may influence the competition for other resources. In competition, apart from the obvious outcomes, such as extinction of one of the competitors or mutual extinction of both of the competing species, a third type of plausible outcome is coexistence. A fundamental problem in ecology lies in unravelling the strategies by which different organisms can continue to ‘coexist’ while competing for limited resources. For symbiosis, in the case of lichens; and for mutualism, like ant–plant mutualism, coexistence involves cooperation [9]. But in the case involving *Parus* sp., where five bird species live within English broad-leaved woodlands, coexistence involves competition [10].

Extensive studies have been performed on intra- and interspecific interactions of microorganisms, including bacteria [4,11,12]. These interactions in bacteria refer to both physical and chemical communication between interacting species. In nature, especially when bacteria struggle for their existence in conditions with limited resources, their cooperation or competition to a large extent depends on their metabolic interactions [12]. Communication between the individuals of a community is made possible through the production, detection and response to an array of chemical signals [13]. Transmission of important information is the key to a successful growing community, whereby the response from the receiver directs the kind of interaction: cooperation or competition. Recent studies have also revealed that bacteria show significant changes in their gene expression profile when confronted with other bacterial species [14,15]. Thus, the challenge is to decipher the behavioural and molecular responses of chemical signals produced and received by bacteria in order to decipher the principles underlying interactions within microbial communities [16,17].

In this paper, we present qualitative and quantitative analyses on interspecific competition between *Bacillus cereus* MSM-S1 and *Pseudomonas* sp. MSM-M1 isolated from the same ecological niche. Experimental data revealed a chemical cross-talk between these two bacterial species, which significantly affected the growth, development and behavioural responses of *B. cereus* MSM-S1 only. Although exploitation of resources is a contributory factor for this antagonism, we find this competition is predominantly driven by the interference of the molecules(s) secreted by *Pseudomonas* sp. MSM-M1 which inhibits the growth of *B. cereus* MSM-S1 as a chemical cue. We also present a mathematical model based on the reaction–diffusion equation, which can be used as a general model to study interspecific competition to demonstrate the interplay between cell concentration and movement, availability of resources and the production and function of secreted inhibitors. Simulations carried out using the model unambiguously confirm the primary role played by the inhibitor in affecting the colony growth dynamics of either species. Specifically, we find that the formation of the inhibition zone between the two bacterial colonies can be attributed to the negative chemotactic effect of the inhibitor on the *B. cereus* MSM-S1 cells thereby modulating the shape of the leading (interacting) edge of *B. cereus* MSM-S1 colony that faces the *Pseudomonas* sp. MSM-M1 colony.

## 2. Material and methods

### 2.1. Isolation and characterization of soil bacteria

Bacteria were isolated from the soil of a service station near Salt Lake (22.58° N, 88.42° E) Kolkata, West Bengal, India. No specific permission was required to collect the soil samples and bacteria from the study site. Soil samples used in this study were collected under the consent of the owner of the service station and this study did not involve endangered or protected species. Isolated bacterial strains were identified by initial biochemical identification methods [18] and by 16S rDNA/rRNA sequencing [19].

### 2.2. Strains and growth media

*Bacillus cereus* MSM-S1 (aerobic, spore forming, Gram positive, rod-shaped, motile and approx. 1 × 3–4 µm in size) and *Pseudomonas* sp. MSM-M1 (aerobic, non-spore forming, Gram negative, rod-shaped, motile and approx. 0.8 × 1.5 µm in size) were used to study interspecific interaction in nutrient broth and semi-solid nutrient agar media (electronic supplementary material).

### 2.3. Studies of bacterial growth pattern in isolation

To measure the growth of *B. cereus* MSM-S1 and *Pseudomonas* sp. MSM-M1 in liquid media, cells were incubated for 16 h in nutrient broth at 30°C with mild shaking and optical density (OD) was measured at 30 min intervals at 600 nm using a Beckman Coulter UV/Vis spectrophotometer (DU^®^730), and OD was plotted against time of incubation.

For monitoring bacterial growth on semi-solid substratum, nutrient agar (0.6%) plates were inoculated by placing 2 µl droplets of bacterial cultures (1 OD) of each strain at the centre of the plate and bacterial colonies were grown at 30°C. Images of bacterial colonies were obtained across different days (to 15 days) using a Bio-Rad Molecular Imager® Gel Doc™ XR System.

All the bacterial cells present in a single colony were released by scraping the cells aseptically from the agar pieces to saline solution. Remaining cells were released further by shaking the agar pieces in saline solution for 15 min at room temperature. Total populations of cells in single colonies of bacteria across different days were obtained by serial plating technique and were expressed in terms of colony forming units (CFU) [20].

### 2.4. Studies of bacterial interaction

To evaluate the effect of the presence of one species of bacteria on the growth of the other, similar experiments were carried out in liquid as well as semi-solid agar media, using co-culture (MSM-M1 and MSM-S1 together) of the isolates where monocultures of MSM-M1 and MSM-S1 served as control and the difference in growth between them was compared. For growth in liquid media, 10^7^ cells of each bacterial species were incubated for 16 h at mild shaking conditions, either as a monoculture or as mixed cultures (in equal proportion) and CFUs ml^−1^ were measured to acquire the nature of the interaction. As MSM-S1 and MSM-M1 give distinctly different colony morphology in nutrient agar plates, it is possible to distinguish colonies of MSM-S1 from that of MSM-M1.

On semi-solid media (0.6% agar) where the movement of bacteria is limited, the experiment was set as a dyadic interaction between *B. cereus* MSM-S1 and *Pseudomonas* sp. MSM-M1. Bacterial cultures were spotted on agar plates maintaining equidistance from the centre along the diameter of the plates. On separate plates, spots of MSM-S1 and MSM-M1 were used as controls in the absence of any interspecific interaction. Bacterial growth on semi-solid media was measured by serial plating methods and expressed in terms of total CFUs present in the colony across different days as compared to the total CFUs in the control colonies.

### 2.5. Confocal laser scanning microscopy

Confocal laser scanning microscopy (CLSM) was performed to observe the morphology and orientation of bacterial cells at the interacting and non-interacting edges of *B. cereus* MSM-S1 and *Pseudomonas* sp. MSM-M1 colonies (see the electronic supplementary material).

### 2.6. Field emission scanning electron microscopy

To gain better resolution and understanding of the differences in the morphology of *B. cereus* MSM-S1 and *Pseudomonas* sp. MSM-M1 at the cellular level, field emission scanning electron microscopy (FESEM) was performed (see the electronic supplementary material).

### 2.7. Inhibition studies

To determine the inhibition activity between the bacterial species, agar pieces were taken from the vicinity of non-interacting edge of the growing colonies of MSM-M1 and MSM-S1 and from the interacting zone between two bacterial colonies and placed on three different places on the growing lawns of isolated *B. cereus* MSM-S1 and *Pseudomonas* sp. MSM-M1.

### 2.8. Statistical analysis

StatistiXL was used to analyse the data, using non-parametric Mann–Whitney *U*-test.

## 3. Experimental results

### 3.1. Isolation and identification of bacterial species

The two soil dwelling bacterial species, confirmed with their phylogenetic positions, were *B. cereus* (Genbank accession no. HM061612) and named *B. cereus* MSM-S1 (NCIM 5361) and *Pseudomonas* sp. (Genbank accession no. GU056312) and named *Pseudomonas* sp. MSM-M1 (NCIM 5360) (see the electronic supplementary material, figure S1’). MSM-S1 and MSM-M1 were chosen to see the effect of antagonistic bacterial interaction as the strains were collected from a shared niche.

### 3.2. Growth characteristics

**Figure 1.**
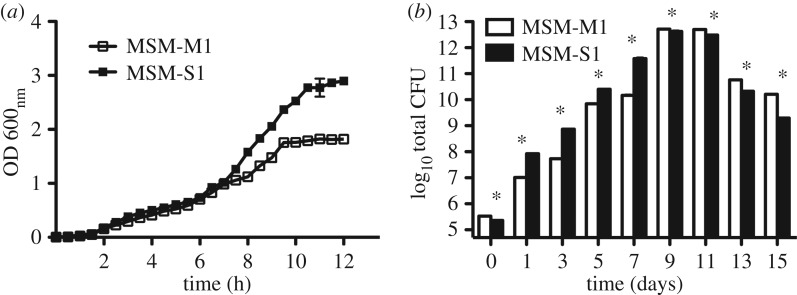
Survivorship graphs of *Bacillus cereus* MSM-S1 and *Pseudomonas* sp*.* MSM-M1 in nutrient-rich conditions. (*a*) Growth characteristics of *Bacillus cereus* MSM-S1 and *Pseudomonas* sp. MSM-M1 in nutrient broth at 0.5 h intervals; (*b*) total CFUs of *Bacillus cereus* MSM-S1 and *Pseudomonas* sp. MSM-M1 on 0.6% nutrient agar plate over 15 days (*U* = 36, d.f._1_ = d.f._2_ = 6, *p* = 0.002). Growth was measured in terms of OD of bacterial culture grown in liquid media and total CFUs were measured in the case of semi-solid agar media. All values are represented as means ± s.d. For both liquid media and nutrient agar plate *n* = 6. Mann–Whitney *U*-test was performed to determine statistical significance.

In liquid culture, the growth of *B. cereus* MSM-S1 was found to be higher than that of *Pseudomonas* sp. MSM-M1. Interestingly, it was observed that both of the bacterial cultures grew equally well until mid-log phase. After this point, MSM-S1 seemed to grow faster than MSM-M1 and eventually became saturated at 2.8 OD, whereas MSM-M1 slowed down its growth past 1 OD and became saturated at 1.5 OD (figure 1*a*).

In the case of semi-solid nutrient media (0.6% agar), the growth of *B. cereus* MSM-S1 was found to be significantly higher than that of *Pseudomonas* sp. MSM-M1 until day 7. Initial concentration of *B. cereus* and *Pseudomonas* sp. cells in one colony was 2.3 × 10^5^ and 3.3 × 10^5^, respectively. On day 7, the total CFUs in each colony of *B. cereus* and *Pseudomonas* sp. were calculated as 3.79 × 10^11^ and 1.46 × 10^10^, respectively (figure 1*b*). After that point, on day 9, both of the bacterial species reached a stationary phase. From day 13 onward, cell numbers started declining for both MSM-S1 and MSM-M1 colonies.

### 3.3. Comparative growth analysis of two competing bacterial isolates

To check the effect of the presence of one species of bacteria on the growth of the other, similar experiments were carried out in both liquid and semi-solid agar media, using a co-culture (*Pseudomonas* sp. MSM-M1 and *B. cereus* MSM-S1) of the isolates, where monocultures of MSM-M1 and MSM-S1 served as controls. The difference in growth yield between them was compared.

**Figure 2.**
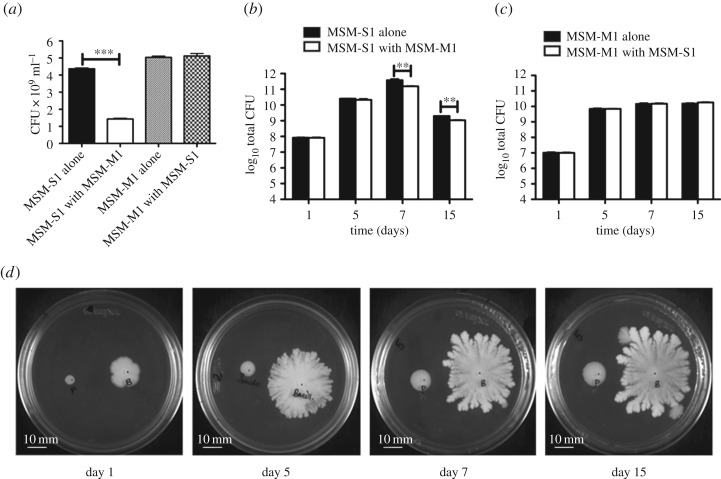
Comparative growth analysis of competing bacterial species. (*a*) Bacterial CFUs of *Bacillus cereus* MSM-S1 produced when grown alone versus *Bacillus cereus* MSM-S1 during interspecific competition, grown in the presence of *Pseudomonas* sp. MSM- M1 after 16 h in nutrient broth (*U* = 36, d.f._1,2_ = 6, *p* = 0.002); CFUs of *Pseudomonas* sp. MSM-M1 produced when grown alone versus *Pseudomonas* sp. MSM-M1 during interspecific competition, grown in the presence of *Bacillus cereus* MSM-S1 after 16 h in nutrient broth. (*b*) Comparison of growth (total CFUs) of *Bacillus cereus* MSM S1 grown alone versus *Bacillus cereus* MSM-S1 grown in the presence of *Pseudomonas* sp. MSM M1 on 0.6% agar plates on day 1 (*U* = 20.5; d.f._1,2_ = 6; *p* = 0.699), day 5 (*U* = 30; d.f._1,2_ = 6; *p* = 0.065), day 7 (*U* = 36; d.f._1,2_ = 6; *p* = 0.002) and day 15 (*U* = 36; d.f._1,2_ = 6; *p* = 0.002). (*c*) *Pseudomonas* sp. MSM-M1 grown alone versus *Pseudomonas* sp. MSM-M1 grown in the presence of *Bacillus cereus* MSM-S1 on 0.6% agar plates on day 1 (*U* = 19; d.f._1,2_ = 6; *p* = 0.937), day 5 (*U* = 19.5; d.f._1,2_ = 6; *p* = 0.937), day 7 (*U* = 20.5; d.f._1,2_ = 6; *p* = 0.699) and day 15 (*U* = 30; d.f._1,2_ =6; *p* = 0.065). All results are shown as means ± s.d. Graphs (*b*,*c*) are represented in log_10_ scale. (*d*) The relative interaction between *Bacillus cereus* MSM-S1 and *Pseudomonas* sp. MSM-M1 on nutrient agar plate across different days. Mann–Whitney *U*-test was performed to determine statistical significance.

The CFUs ml^−1^ of *B. cereus* MSM-S1 was significantly reduced (approx. threefold) in the presence of *Pseudomonas* sp. MSM-M1 (1.4 × 10^9^) compared to the corresponding monoculture (4.36 × 10^9^). To the contrary, the CFUs of *Pseudomonas* sp. MSM-M1 were not altered significantly in the presence (5.03 × 10^9^) or the absence (5.23 × 10^9^) of *B. cereus* MSM-S1 in the growth medium (figure 2*a*).

To assess whether the two bacterial species competed with each other in a similar manner on a semi-solid biotic surface, we performed parallel experiments in nutrient media containing 0.6% agar. Here, similar growth patterns were observed for co-cultures. Plates were incubated for 15 days and the total number of bacteria present in each colony (CFU) was measured at different time points (days 1, 5, 7 and 15) and were compared to the growth of single bacterial colonies (grown in the absence of the competing species) at same time intervals. For *B. cereus* MSM-S1 colonies, no significant growth difference was found on days 1 and 5, but interestingly, from day 7 onward the growth and pattern of *B. cereus* MSM-S1 colonies were altered significantly in the presence of *Pseudomonas* sp. MSM-M1 (figure 2*b*,*d*). Substantial reduction of MSM-S1 colony movement was observed in the interacting zone between *Pseudomonas* sp. MSM-M1 and *B. cereus* MSM-S1, where a zone of inhibition appeared between the two colonies. This resulted in a concave shape of the *B. cereus* MSM-S1 colony at the interacting edge (figure 2*d*). For *Pseudomonas* sp. MSM-M1, no significant difference in the growth and pattern (as mono- or co-culture) was observed, for all five time points mentioned (figure 2*c*,*d*).

### 3.4. Comparative analyses of orientation and morphology of cells of two competing bacterial colonies

**Figure 3.**
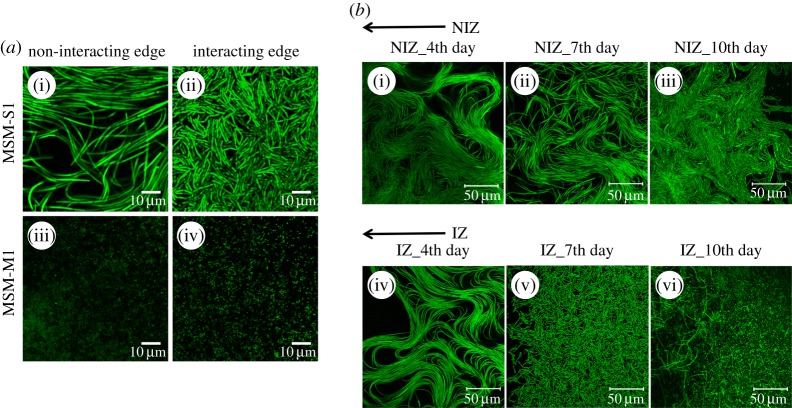
CLSM studies of cellular orientation and morphology during interspecific interaction. (*a*) CLSM images of the non-interacting and the interacting edges of *Bacillus cereus* MSM-S1 (*a*(i)(ii)) and *Pseudomonas* sp. MSM-M1 (*a*(iii)(iv)) colonies grown for 7 days. (*b*) CLSM studies of non-interacting (*b*(i–iii)) and interacting edges of *Bacillus cereus* MSM-S1 (*b*(iv–vi)) colonies. All the figures represented here are stacked in *z*-axis.

CLSM was performed to understand the morphology and orientation of bacterial cells, both at interacting and non-interacting edges of *B. cereus* MSM-S1 and *Pseudomonas sp.* MSM-M1 colonies. Confocal microscopic images of 7 day grown colonies during interspecific interaction clearly showed contrasting differences in cellular organization and cell morphology between the interacting and non-interacting edges of *B. cereus* MSM-S1. Upon evaluation of the non-interacting edge of MSM-S1, the cells appeared oriented and associated as elongated chains, which was a signature of swarming cells (figure 3*a*(i)). On the other hand, at the interacting edge of MSM-S1, cells appeared disorganized and relatively smaller in size than those of non-interacting edge, which indicated that the cells changed their orientation randomly in order to avoid unfavourable conditions (figure 3*a*(ii)). For *Pseudomonas* sp. MSM-M1, no difference in morphology and orientation was observed between the cells of non-interacting and interacting edges of growing colonies (figure 3*a*(iii)(iv)).

To emphasize the changes that were observed during 7th day of interspecific interaction at the interacting edge of *B. cereus* MSM-S1, a day wise study of *B. cereus* MSM-S1 was performed. A prominent change was observed at the interacting edge of *B. cereus* MSM-S1 which indicated that the homogeneity of bacterial arrangement was lost with time. On the 4th day of interaction, both the edges showed similar kind of arrangement of cells indicating cell swarming (figure 3*b*(i)(iv)), but from day 7 onwards the cells at the interacting edge were found to have lost the homogeneity in terms of morphology and orientation and appeared as clustered and randomly distributed in the case of interacting edge (figure 3*b*(ii)(v)). Moreover, the appearance of endospores was also evident in the population of cells at the interacting edge, indicating a response to unfavourable condition induced by the inhibitor secreted by *Pseudomonas* sp. On day 10, most of the cells at the interacting edge formed spores due to stress from interspecific interaction (figure 3*b*(iii)(vi)). Although at the non-interacting edge, on days 7 and 10, a few cells appeared stressed, perhaps due to nutrient depletion at the local level, but overall orientation of cells was similar to that of cells grown for 4 days.

**Figure 4.**
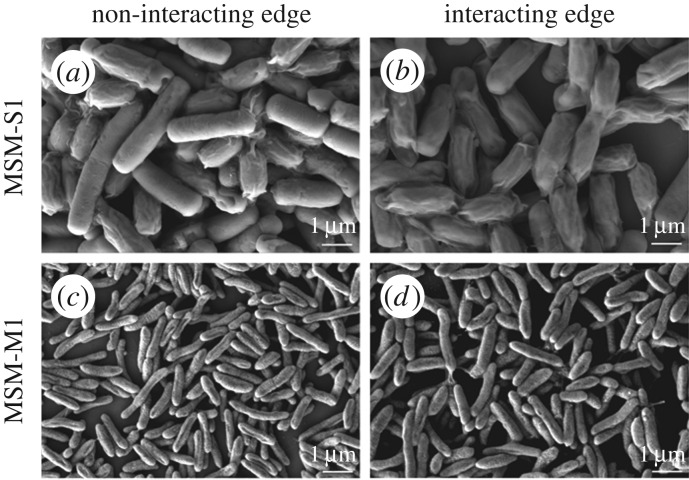
FESEM studies of cellular level interactions between *Bacillus cereus* MSM-S1 and *Pseudomonas* sp. MSM-M1. FESEM images of the non-interacting and the interacting edges of *Bacillus cereus* MSM-S1 (*a*,*b*) and *Pseudomonas* sp. MSM-M1 (*c*,*d*).

To gain better insight into the differences in the cellular morphology of *B. cereus* MSM-S1 and *Pseudomonas* sp. MSM-M1 at the interacting and non-interacting edges FESEM was performed. At the interacting edge, MSM-S1 cells appeared to be under stressful conditions as a considerable number of the *B. cereus* MSM-S1 cells had formed spores (figure 4*b*). On the other hand, at the non-interacting edge, the number of stressed cells was substantially lower compared to the interacting edge (figure 4*a*). However, no cellular stress was observed in the cells of MSM-M1, either at the interacting or non-interacting edges of colonies grown until day 7 (figure 4*c*,*d*).

All of these observations raised the question about the contributory factor(s) behind the appearance of the zone of inhibition between the growing colonies of *B. cereus* MSM-S1 and *Pseudomonas* sp. MSM-M1. It could be an effect of either (i) nutrition depletion in the zone between two growing colonies or (ii) the presence of inhibitory molecule(s) produced and secreted by MSM-M1 as a mechanism of interference.

### 3.5. Mechanism of interference

**Figure 5.**
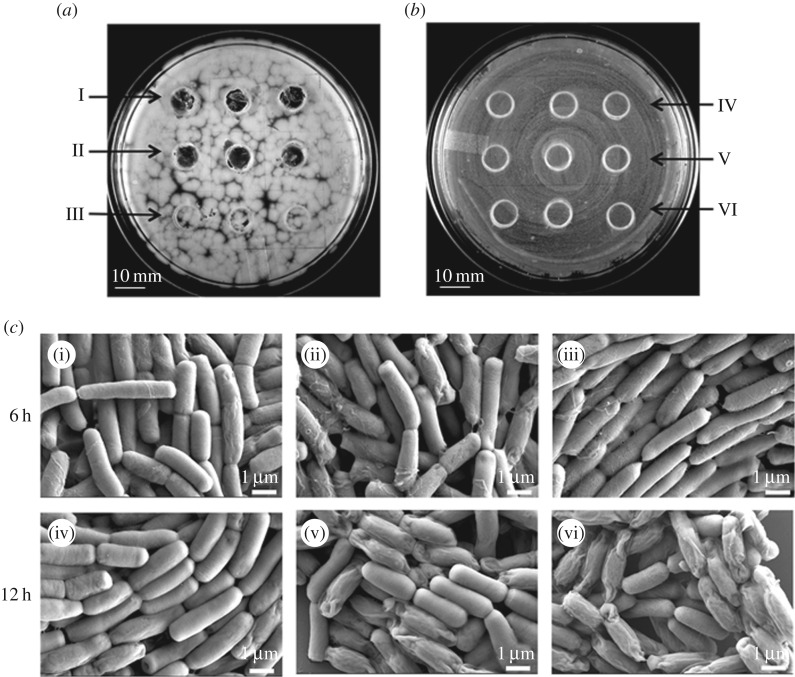
Studies on the role of inhibitor for the mechanism of interference. Agar pieces taken (in triplicates) from the regions close to growing colonies of MSM-M1 (I and IV) and MSM-S1 (III and VI) and from the inhibition zone between MSM-M1 and MSM-S1 (II and V) were placed over (*a*) a lawn of *Bacillus cereus* MSM-S1 and over (*b*) a lawn of *Pseudomonas* sp. MSM M1. (*c*) Induction of sporulation in *Bacillus cereus* (after 6 and 12 h) by agar pieces taken from control plate (i) and (iv), from the regions close to grown colonies of MSM-M1 (ii) and (v) and from the inhibition zone between MSM-M1 and MSM-S1 colonies (iii) and (vi).

To address the above questions, agar pieces were taken from the vicinity of growing colonies of *Pseudomonas* sp. MSM-M1, *B. cereus* MSM-S1 and from the interacting zone between the two bacterial colonies and placed on three different places on the growing lawns of *B. cereus* MSM-S1 and *Pseudomonas* sp. MSM-M1. The agar pieces taken from the inhibition zone inhibited the growth of *B. cereus* MSM-S1. Interestingly, agar pieces taken from the area around *Pseudomonas* sp. MSM-M1 colony also inhibited the growth of MSM-S1 in the same manner. Whereas, agar pieces close to the non-interacting edge of the *B. cereus* MSM-S1 colony did not exert any such inhibition (figure 5*a*). No inhibition of MSM-M1 was observed by placing similar agar pieces on the growing lawn of *Pseudomoas* sp. MSM-M1 (figure 5*b*). Thus, these results confirmed that the observed growth inhibition of *B. cereus* MSM-S1 in co-culture was mediated by the secreted chemical(s) of *Pseudomonas* sp. MSM-M1. This chemical could be a generic inhibitor that impacts the growth of its competitors or it could be a metabolite of *Pseudomonas* sp. which is being sensed by *B. cereus* as a chemical cue. However, it is clear that secretion of the inhibitory molecules by *Pseudomonas* sp. MSM-M1 was irrespective of the presence or the absence of *B. cereus* MSM-S1.

Furthermore, to observe the effect of the inhibitor on the induction of sporulation in *B. cereus* MSM-S1, agar pieces containing the inhibitor were placed on the growing lawn of MSM-S1. Scanning electron microscopy was performed on *B. cereus* MSM-S1 cells that were exposed for 6 and 12 h post inhibition (figure 5*c*). Until 6 h of incubation in the presence of the inhibitor no spore was observed, whereas after 12 h of incubation, the appearance of endospores (43–46%) was evident in *B. cereus* MSM-S1.

Thus, all the above experimental results confirmed the presence of the inhibitor secreted by *Pseudomonas* sp. MSM-M1 in its surrounding media which negatively regulates the growth and movement of *B. cereus* MSM-S1 cells and colony.

### 3.6. Reaction–diffusion model of colony growth

We developed a computational model to better understand the precise mechanism by which the inhibitor affects the dynamics of colony growth. We were specifically interested in exploring the effect of the inhibitor on the formation of a prominent inhibition zone between the two bacterial colonies, which is observed in experiments. Our model was informed by experimental observations described in earlier sections and is based on a set of coupled reaction–diffusion equations [21,22] that describe the spatio-temporal dynamics of the two bacterial colonies, the inhibitor and nutrients. The model is adapted from generic models of pattern formation [23–28] used to study cooperative as well as antagonistic growth dynamics of one or more bacterial colonies in the presence of regulatory chemical species. As the colony growth is mostly planar, we employ a two-dimensional spatial model described by the following set of coupled partial differential equations (PDEs):

3.1 $$\begin{array}{rl} \frac{\partial u_{1}}{\partial t} & =a_{1}u_{1}g(1-e^{-u_{1}})(1-\eta_{1}h_{2})-d_{1}u_{1}+\exp\left(-\frac{h_{2}}{hc_{2}}\right)(\Gamma_{1}\nabla^{2}u_{1})+c_{12}\nabla u_{1}\cdot \nabla h_{2}, \end{array}$$

3.2 $$\begin{array}{rl} \frac{\partial u_{2}}{\partial t} & =a_{2}u_{2}g(1-e^{-u_{2}})(1-\delta_{2}h_{2})-d_{2}u_{2}+\Gamma_{2}\nabla^{2}u_{2}, \end{array}$$

3.3 $$\begin{array}{rl} \frac{\partial h_{2}}{\partial t} & =u_{2}(1-\lambda_{2}h_{2})(1-e^{-g})+\gamma_{2}\nabla^{2}h_{2} \end{array}$$

3.4 $$\begin{array}{rl} \;\mathrm{and}\;\frac{\partial g}{\partial t} & =-g\sum_{i=1}^{2}u_{i}+\nabla^{2}g. \end{array}$$

**Figure 6.**
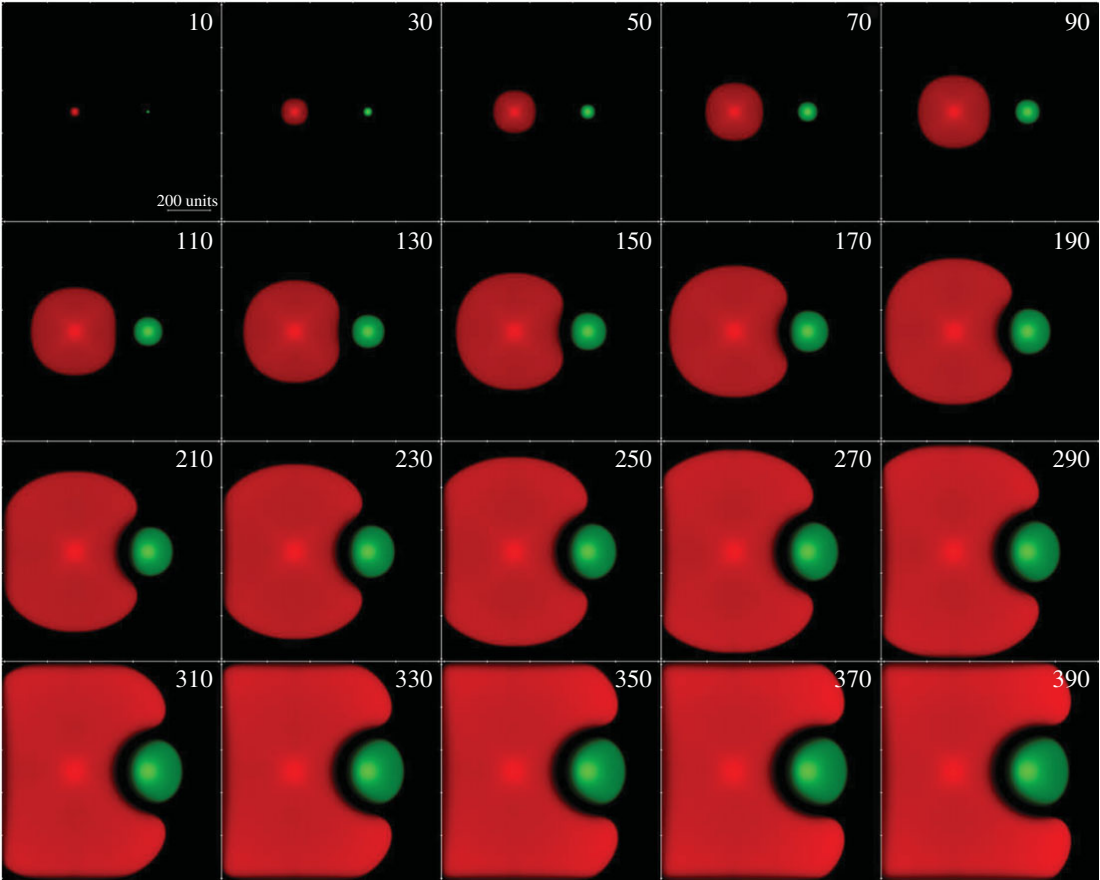
Colony growth over time. Time lapse images of colony growth dynamics. Images were generated based on simulations. Parameters used: *a*_1_ = 0.8, *a*_2_ = 0.2, *h_c_*_2_ = 0.5, *Γ*_1_ = 0.01, *Γ*_2_ = 0.005, *γ*_2_ = 0.39, *λ*_2_ = 1, *δ*_2_ = 0.01, *d*_1_ = 0.001, *d*_2_ = 0.0001, *d_h_*_2_ = 0, *c*_12_ = 0.4, *η*_1_ = 0.4.

Here $u_{1}(x,y;t)$ and $u_{2}(x,y;\;t)$ represent the densities of *B. cereus* MSM-S1 and *Pseudomonas* sp. MSM-M1, respectively, $h_{2}(x,y;\;t)$ represents the density of the inhibitor released by *Pseudomonas* sp. MSM-M1 and g(x,y; t) is the nutrient density. *Bacillus cereus* MSM-S1 does not produce any inhibitor which affects either its own dynamics or that of *Pseudomonas* sp. MSM-M1. Here *a*_1_ and *a*_2_ are the maximum growth rates of S1 and M1, respectively. To account for the fact that the colony density changes much more rapidly near the boundary of the colony than in the interior [29–31] where it can be assumed to increase linearly, the additional density-dependent factor $\left(1-e^{-u_{i}}\right)$ was introduced in the growth rate term. The growth rate of S1 is negatively regulated by the presence of the inhibitor and this occurs with rate *η*_1_. We also assume that the presence of the inhibitor slows down the diffusion rate of *B. cereus* MSM-S1, and this effect is manifest through the modulation of the diffusion coefficient *Γ*_1_ by an exponential factor $\exp(-(h_{2}/hc_{2})),$ which depends on the density of the inhibitor. However, the inhibitor has no effect on the motility of *Pseudomonas* sp. MSM-M1 which diffuses with the rate *Γ*_2_. *Pseudomonas* sp. MSM-M1 incurs a certain cost in producing the inhibitor as a result of which its growth rate may be affected. The cost of inhibitor production on the growth rate of M1 is taken into account through the term $\left(1-\delta_{2}h_{2}\right)$ in equation (3.2). In addition to the diffusive (random) component of cell motility, there can be a chemotactic (directed) component that is manifest in response to the presence of a chemical stimulus in the environment which in our system is secreted by the competing species [32,33]. The last term on the right-hand side of equation (3.1) represents the chemotactic effect resulting from interaction between the inhibitor released by *Pseudomonas* sp. MSM-M1 and *B. cereus* MSM-S1. The sign of the term indicates that we are invoking negative chemotaxis wherein the *B. cereus* MSM-S1 cells try to move away from a region of higher inhibitor concentration to a region of lower inhibitor concentration with the coefficient *c*_12_ representing the strength of the chemotactic interaction. The inhibitor has no effect on the motility of *Pseudomonas* sp. MSM-M1. Here *d*_1_ and *d*_2_ represent the death rates of the *B. cereus* MSM-S1 and *Pseudomonas* sp. MSM-M1 cells, respectively. The inhibitor after being secreted by the *Pseudomonas* sp. MSM-M1 cells diffuses in the two-dimensional plane with rate *γ*_2_ and *λ*_2_ represents the inhibition rate of inhibitor production. The equations are written in terms of dimensionless variables. Each bacterial colony was seeded initially by specifying a non-zero initial density at spatial lattice points having the same *y*-coordinate but separated along the *x*-direction by *N*/3 lattice points where *N* is the size of the square lattice. The initial distribution of S1 and M1 densities was symmetric about the *y*-axis. The nutrients were initially uniformly distributed throughout the lattice while the initial density of the inhibitor is set to zero. (See electronic supplementary material for details.) The above set of coupled PDEs were solved numerically [34,35] subject to the initial conditions specified above. Figure 6 shows the gradual growth of the two colonies over time when the chemotactic coefficient *c*_12_ is non-zero. As the two colonies grow, they initially maintain a spherical shape but gradually the presence of the inhibitor starts distorting the shape of the front of *B. cereus* MSM-S1 (red) colony moving towards the *Pseudomonas* sp. MSM-M1 (green) colony. The figure clearly shows the eventual formation of a sharply defined inhibition zone between the two colonies, which is consistent with experimental observations (figure 2*d*). The width of the inhibition zone becomes nearly constant, a result that is also consistent with the experimental observations depicted in figure 2*d*. If the growth rates (*a*_1_ and *a*_2_) of S1 and M1 are assumed to vary randomly (within a specified range) across the colony due to heterogeneity in nutrient distribution, the qualitative nature of the colony growth pattern changes from smooth (figure 6) to the more realistic slightly rough pattern (see the electronic supplementary material, figure S3) as can be seen when the latter is compared to the experimental images (figure 2*d*) of colony growth. However, the formation of the inhibition zone and its width remain unaffected.

**Figure 7.**
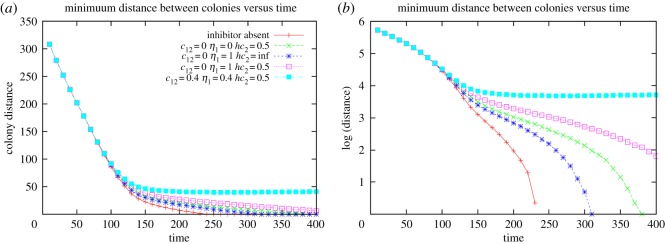
Minimum distance between colonies. Variation of minimum distance (*a*) and log(minimum distance) (*b*) between the two colonies with time. Parameters used are the same as in figure 6. Figure was generated based on simulations.

To verify that the chemotactic effect is primarily responsible for the formation of the inhibition zone, we plotted the temporal variation of the minimum distance between the two colonies (see the electronic supplementary material, section ‘Minimum gap calculation’ and figure S2) under various conditions (figure 7). In the absence of the inhibitor, the two colonies eventually overlap as is evident from the fact that the minimum distance between the colonies goes to zero (red curve in figure 7). This clearly indicates that nutrient depletion alone cannot explain the formation of the inhibition zone in the absence of the inhibitor. Moreover, in the absence of chemotaxis (*c*_12_ = 0), neither inhibition of the growth rate (blue dashed line) nor inhibition of the diffusion rate (green dashed line) nor a combination of these two factors (dotted magenta line) is able to account for the formation of the inhibition zone. This is evident from the fact that the minimum distance goes to zero, albeit more slowly, even when inhibition of growth rate as well as diffusion rate of S1 are taken into account (see green, blue and magenta curves in figure 7). Only when $c_{12}\neq 0$ do we find the appearance of an inhibition zone (cyan curve in figure 7) of nearly constant width. The increase in the minimum distance between the colonies on increasing the chemotactic coefficient is shown in the electronic supplementary material, figure S4. These results highlight the primary role of inhibitor induced negative chemotaxis in producing the observed pattern seen in experiments.

**Figure 8.**
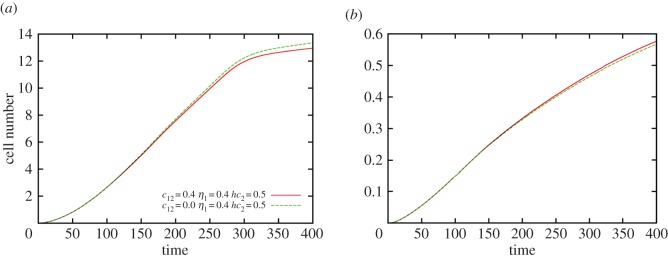
Temporal variation of cell population. Variation in cell number of the (*a*) red (S1) and (*b*) green (M1) colonies with time. Parameters used are the same as in figure 6. Figure was generated based on simulations.

The total number of cells of the *i*th species was obtained by integrating the cell density over the area element d*x*d*y* to give $\Omega_{i}(t)=\int \int dx\;dyu_{i}(x,y;\;t)$. Figure 8 shows how the populations of the red and green colonies are affected in the presence and absence of the negative chemotactic effect induced by the inhibitor. When *c*_12_ ≠ 0, the total number of cells in the red colony (S1) decreases slightly over time (figure 8) compared with the case when the chemotactic effect is absent. By contrast, the total number of cells in the green colony (M1) increases slightly over time under the same conditions. This is also evident from the decrease in the total number of S1 cells and increase in the total number of M1 cells with increase in chemotactic coefficient (see the electronic supplementary material, figure S5). This observation can be attributed to the formation of the inhibition zone which prevents further expansion of the leading edge of the red (S1) colony restricting access of the *B. cereus* MSM-S1 cells making up the leading edge to nutrients in the inhibition zone. This forces the *B. cereus* MSM-S1 cells near the leading edge to compete for dwindling nutrients thereby reducing the rate at which they can divide and increase their population. Whereas, the *Pseudomonas* sp. MSM-M1 cells in the leading edge of the green (M1) colony have to encounter lesser competition for nutrients due to the absence of *B. cereus* MSM-S1 cells in the inhibition zone. As a consequence, their population increases slightly when the negative chemotactic effect is taken into account.

## 4. Discussion

In this study, we examine how a slower growing bacterial species can survive being outcompeted by a faster growing competitor belonging to a taxonomically unrelated strain, when both inhabit the same ecological niche. The most prominent observed changes in mixed cultures when compared with mono-cultures were: (i) reduction in the growth of the faster growing strain *B. cereus* MSM-S1 in both nutrient broth and on nutrient agar plates and (ii) the appearance of a zone of inhibition between two growing colonies of *Pseudomonas* sp. MSM-M1 and *B. cereus* MSM-S1 on semi-solid nutrient agar plates along with modulation of the shape of the interacting edge of the S1 colony.

Our results imply that the observed growth reduction in MSM-S1 can be attributed to the inhibitor produced by *Pseudomonas* sp. MSM-M1. This was apparent from the co-culture experiments in liquid and semi-solid cultures and further confirmed by the appearance of an inhibition zone on a growing lawn of *B. cereus* MSM-S1 (figure 4*a*). Thus, the results supported a competitive strategy adopted by *Pseudomonas* sp. MSM-M1 based on interference (through production of antimicrobial compound/metabolite) while no such strategy is evident for *B. cereus* MSM-S1. As a consequence, M1 is able to survive the competition with S1 leading to the coexistence of the two competitive bacterial species. In a different study, a similar case of interference by *Pseudomonas* sp. A21 on *Pedobacter* sp. V48 was reported by Garbeva & Boer [14].

A study involving pathogenic *Pseudomonas aeruginosa* and *Burkholderia cepacia* by Eberl *et al.* demonstrates a unique mechanism of cell–cell communication driven by *N*-acyl-homoserine lactone. Their interaction is an indispensable aspect in the pathogenicity of cystic fibrosis [36]. During interspecific competition between *Pseudomonas fluorescens* Pf0-1 and *Bacillus* sp. V102, *Pseudomonas fluorescens* Pf0-1 is unable to restrain the growth of *Bacillus* sp. V102 [20]. Interestingly, Powers *et al.* [37] showed inhibition of cell differentiation in *Bacillus subtilis* NCIB 3610 by inhibitor molecule produced by *Pseudomonas protegens* pf-5. In our study, *Pseudomonas* sp. MSM-M1 negatively influenced the growth of *B. cereus* MSM-S1 and also the inhibitor produced by the *Pseudomonas* sp. could induce cellular differentiation (spore formation) in *B. cereus* MSM-S1. These contrasting outcomes indicate that bacteria within the same genera can employ diverse competitive strategies to survive and coexist with different bacterial species in different ecosystems.

Further studies involving CLSM and FESEM established the primary role played by the inhibitor secreted by *Pseudomonas* sp. MSM-M1 in the growth, cell division and motility of *B. cereus* MSM-S1. While a prominent change in the cellular orientation and cell morphology of *B. cereus* MSM-S1 was observed in the interacting edge compared to non-interacting edge on day 7, a time lapse study revealed the progression of cellular changes in *B. cereus* MSM-S1 with time. On day 4, while the cells at both interacting and non-interacting edges showed homogeneity in their branching pattern (as associated chains), the homogeneity was lost in the interacting edge over time. Moreover, FESEM images clearly depicted the heterogeneous cell population at the interacting edge, as 40–50% of *B. cereus* MSM-S1 cells formed endospores due to the presence of inhibitor produced by *Pseudomonas* sp. MSM-M1.

To validate our experimental results and determine the precise mechanism by which the inhibitor affects the colony growth patterns, a computational model based on the reaction–diffusion mechanism was developed. Our simulation results revealed that the three important factors involved in affecting the pattern and dynamics of colony growth are cell motility and cell division dynamics, fluctuating nutrient distribution and inhibitor dynamics. Negative chemotaxis in *B. cereus* MSM-S1 due to the presence of inhibitor released by *Pseudomonas* sp. MSM-M1 was found to be solely responsible for the formation of the inhibition zone. The *B. cereus* cells preferred to move down the inhibitor gradient thereby avoiding direct interaction with the *Pseudomonas* sp. MSM-M1 colony. In the absence of negative chemotaxis, no inhibition zone was observed.

Thus, on the basis of our experimental and computational modelling studies, it was apparent that *Pseudomonas* sp. MSM-M1, being a slow grower in our experimental set-up, employed a strategy involving secretion of inhibitor(s) to repel (and thereby prevent being outcompeted by) the faster growing *B. cereus* MSM-S1. On the contrary, *B. cereus* MSM-S1 underwent stress due to the presence of secreted inhibitor/metabolite by the competing *Pseudomonas* sp. MSM-M1. Initially, at a lower concentration of inhibitor, MSM-S1 preferred to move away from MSM-M1, but eventually, when the cells experienced the threshold concentration of inhibitor, MSM-S1 stopped growing further and differentiated into spores at the interacting edge of the colony. This stress response was evident from our confocal and SEM analyses (figures 3 and 4). Similar antagonism by a *Pseudomonas fluorescens* strain towards a *B. cereus* strain was reported by Simoes *et al.* [11], where it was observed that under iron-limitation, *Pseudomonas fluorescens* secreted an antagonistic metabolite which inhibited the growth and induced sporulation in *B. cereus* cells when cultured together.

We use the word inhibitor to describe the secreted chemical that inhibits the growth and advance of the *B. cereus* MSM-S1 colony. As the two species inhabit the same ecological niche, it is quite possible that the inhibitor released by *Pseudomonas* sp. MSM-M1 arose as an evolutionary response that enables it to successfully compete against the faster growing MSM-S1 species. However, the set of experiments (both experimental and computational) described here cannot rule out the possibility that the inhibitor is just a metabolic by-product released by MSM-M1 which acts as a chemical cue that negatively regulates the growth and spread of MSM-S1 colony. Even if the latter is true, our analysis, which successfully explains the colony growth patterns and the appearance of an inhibition zone, remains valid and the model described in this study will inform the future studies on interspecific competitions in bacterial communities.

In conclusion, this study revealed an interesting interplay between two competing bacterial species of different genera that highlights the primary role of inhibitor mediated negative chemotaxis of competing species (*B. cereus* MSM-S1) in the survival of the *Pseudomonas* sp. MSM-M1 colony. Similarly, the decision by *B. cereus* MSM-S1 to form spores in adverse conditions brought about by the presence of the inhibitor is also suggestive of a survival strategy. Future studies on purified inhibitor and gene expression profiles of competing *B. cereus* MSM-S1 and *Pseudomonas* sp. MSM-M1 will further advance the understanding of these bacterial survival strategies.

## Supplementary Material

Supporting Information: contains supplementary or supporting methods and figures

## Data accessibility

The 16S ribosomal DNA sequences for the study organisms were deposited to Genbank (NCBI). The Genbank accession number for *B. cereus* is HM061612 (https://www.ncbi.nlm.nih.gov/nuccore/HM061612) and for *Pseudomonas* sp. is GU056312 (https://www.ncbi.nlm.nih.gov/nuccore/GU056312). Additional data pertaining to this paper are deposited in Dryad: http://dx.doi.org/10.5061/dryad.vh810 [38].
