# Supplementary material for: Deciphering a survival strategy during the interspecific competition between Bacillus cereus MSM-S1 and Pseudomonas sp. MSM-M1
Source: R Soc Open Sci. 2016 Nov 23;3(11):160438. doi: 10.1098/rsos.160438 (PMC5180123; doi:10.1098/rsos.160438)
Supplement: Supporting Information: contains supplementary or supporting methods and figures [file rsos160438supp1.doc]

**Supporting Information on Methods, Results and Reaction-Diffusion Model**

**Supplementary methods**

**Strains and growth media**

*Bacillus cereus* MSM-S1 (aerobic, spore forming, Gram positive, rod-shaped, motile and ~1 X 3-4µm in size) and *Pseudomonas* sp. MSM-M1 (aerobic, non-spore forming, Gram negative, rod-shaped, motile and ~0.8 X 1.5µm in size*)* were used to study interspecific interaction. For liquid cultures, bacteria were inoculated from the frozen stock and were grown in nutrient broth for 16 hrs at 30°C with mild shaking. Bacterial cultures of 1 OD @ 600 nm correspond to 1.4 X 10^8^ and 2 X 10^8^ cells per millilitre of MSM-S1 and MSM-M1, respectively. For growth on semi-solid biotic surface, bacteria were grown on nutrient media containing 0.6% agar.

**Confocal Laser Scanning Microscopy**

Confocal laser scanning microscopy was performed, both at interacting and non-interacting zones of *Bacillus cereus* MSM-S1 and *Pseudomonas sp.* MSM-M1. Cells were initially incubated on 0.6% nutrient agar for 7 days and the colonies were cut into equal sized cubes. Further, the cells in the colonies were stained with 0.005% acridine orange (w/v). Acridine orange stains the bacteria and represents cell structure and cellular association. The cells were then washed with 1X PBS. Finally, they were placed on coverglass bottom dishes (Genetix, 200350) and were scanned under CLSM-710 (Axio Observer Microscope, Version Z.1; Carl Zeiss, Germany). The image acquisition was completed through Zen 2010 software using Plan Apochromat 100X oil immersion objective with numerical aperture 1.4. The samples were excited at 488nm using argon laser and emission of 493 to 576 nm was detected. Images of 8-bit depth having a frame size of 512X512 were maintained throughout the experiment.

**Field Emission Scanning Electron Microscopy (FESEM)**

In order to get better resolution and understanding of the observed differences at the cellular level, Field Emission Scanning Electron Microscopy was performed on *Bacillus cereus* MSM-S1 and *Pseudomonas* sp. MSM-M1 from interacting and non interacting zones**.** Cells were first grown on 0.6% nutrient agar for 7 days and then scrapped-off of the margin of growing colonies and fixed with 200 µl of 2.5% glutaraldehyde solution (in 1X PBS) for 1 hour in dark at room temperature. After that, cells were re-suspended in 200 µl of 0.1% OsO_4_ and kept for 30 minutes at room temperature. After repeated wash with 1X PBS, cells were resuspended in 30%, 50%, 70%, 90% and 100% alcohol respectively. Finally, cells were taken in 20 µl of 100% ethanol and from there 5 µl of cells were put on single side polished silicon wafers (Sigma-Aldrich, 646687). The samples were dried using desiccators and were coated with a thin layer of conducting metal, specifically, gold-palladium in sputter coater (Quorum Technologies Ltd.) before SEM. Samples were observed under Supra 55 VP, Scanning electron microscopy (Carl Zeiss) using SmartSEM software [1, 2].


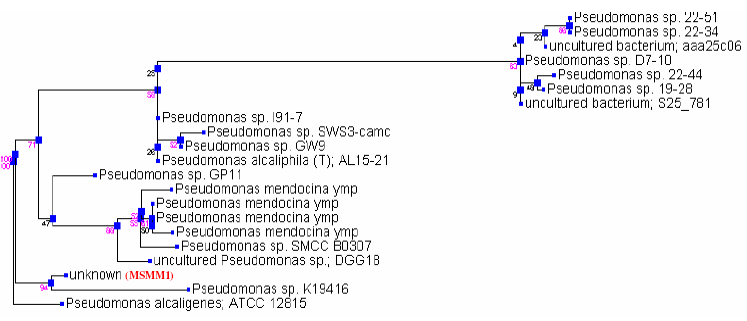
**Supplementary Figure S1:**

S1

**
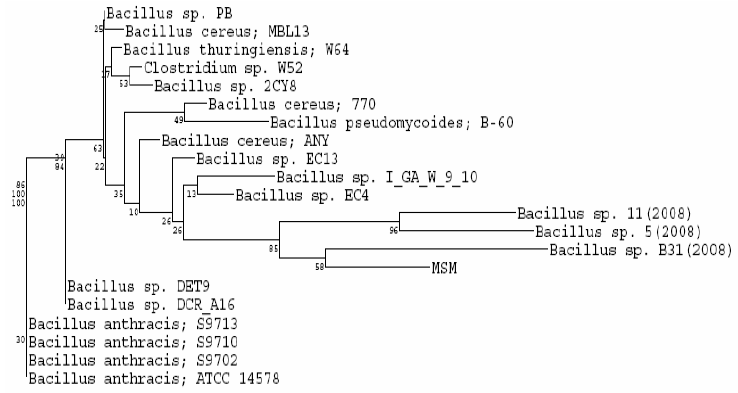
**

**Supplementary Figure S1. Phylogenic dentification of isolated MSM-M1 and MSM-S1:** Sequencing of 16s ribosomal DNA amplified by Polymerase Chain Reaction of isolated genomimc DNA from MSM-M1 and MSM-S1 and phylogenic analysis of the PCR product revealed that the strains are closely associated with *Pseudomonas sp.* and *Bacillus cereus,*  respectively.

**Supplementary information for Reaction-Diffusion Model**

**Numerical Solution of the coupled PDE’s**

The numerical solutions for the coupled PDEs are obtained using the finite difference method. To approximate the model equations by finite differences we divide the closed domain by a set of lines parallel to the spatial and time axes to form a grid or a mesh. We shall assume, for simplicity, that the sets of lines are equally spaced such that the distance between grid points is dx=D and dt respectively. A grid of size 512×512 was used with *D*= 0.2 and *dt* = 0.01. The results critically depends on the value of λ= dt/D^2^. The numerical solution is stable only for sufficiently small λ. The scheme is thus said to be conditionally stable. We restricted the value of λ < 0.5 for numerical stability of the system. The initial values of the nutrient field, inhibitor density and the initial concentrations of the two bacterial species were chosen as follows. u_1_(x=N/3, y=N/2; t=0) = u_2_(x=2N/3,y=N/2;t=0)=1. At all other grid points, the initial bacterial concentrations are set to zero. g(x, y; t=0) = 8 and h_2_(x, y; t=0) = 0 for all x,y. Neumann boundary conditions are used to evolve the system. In the finite difference method, the simplest difference scheme based at the grid point (x; t) uses a forward difference for the time derivative, i.e.

$$u_{i}(x;t+dt)=u_{i}(x;t)+dt\frac{\partial u_{i}(x;t)}{\partial t}$$

For computing Laplacian, we use:

$$\nabla^{2}u(x,y)=\theta\frac{u(x+1,y)+u(x-1,y)+u(x,y+1)+u(x,y-1)-4u(x,y)}{D^{2}}$$

$$+(1-\theta)\frac{u(x+1,y+1)+u(x-1,y+1)+u(x+1,y-1)+u(x-1,y-1)-4u(x,y)}{D^{2}}$$

where θ can be chosen to lie in the (0,1) interval. Since our system has a circular symmetry we chose θ to be 0.5. Similarly for the single spatial derivative we use

$$\frac{\partial u}{\partial x}(x,y)=\theta\frac{(u(x+1,y)-u(x-1,y))}{2D}$$

$$+(1-\theta)\frac{u(x+1,y+1)+u(x+1,y-1)-u(x-1,y+1)-u(x-1,y-1)-4u(x,y)}{2D}$$

**Minimum gap calculation**

In order to calculate the minimum gap, we first find the border of both the colonies (pink in case of S1/red colony and cyan in case of M1/green colony in Fig. S3). For finding the border, we need to select an intensity threshold (in our case it is 16/256) which is based on how bright a particular location is (for example the red colony can take values from 0 to 256 and we impose the condition that any point which has value more than 16 belongs to the colony) and then determine the border. Once the borders are determined, we find the minimum distance between pink and cyan regions.


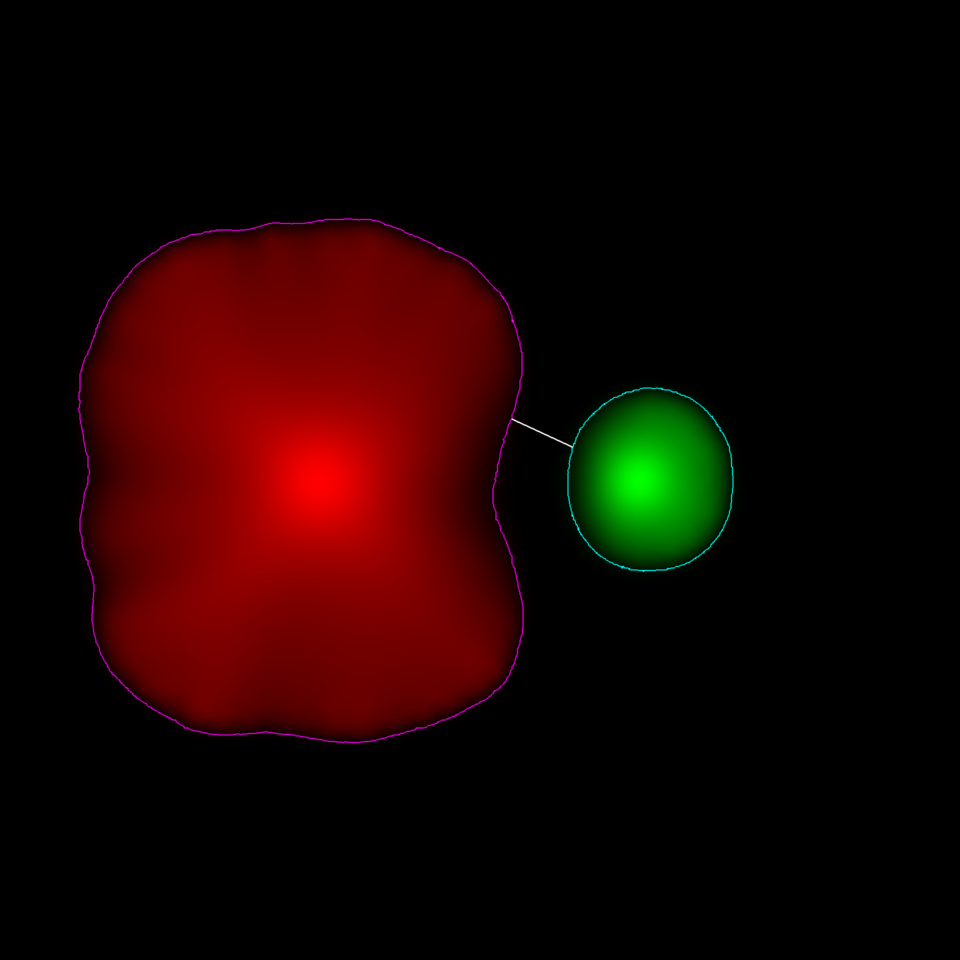


**Figure S2**. **Colony boundaries**. The boundary of the red (S1) colony (shown in pink) and green (M1) colony (shown in cyan).


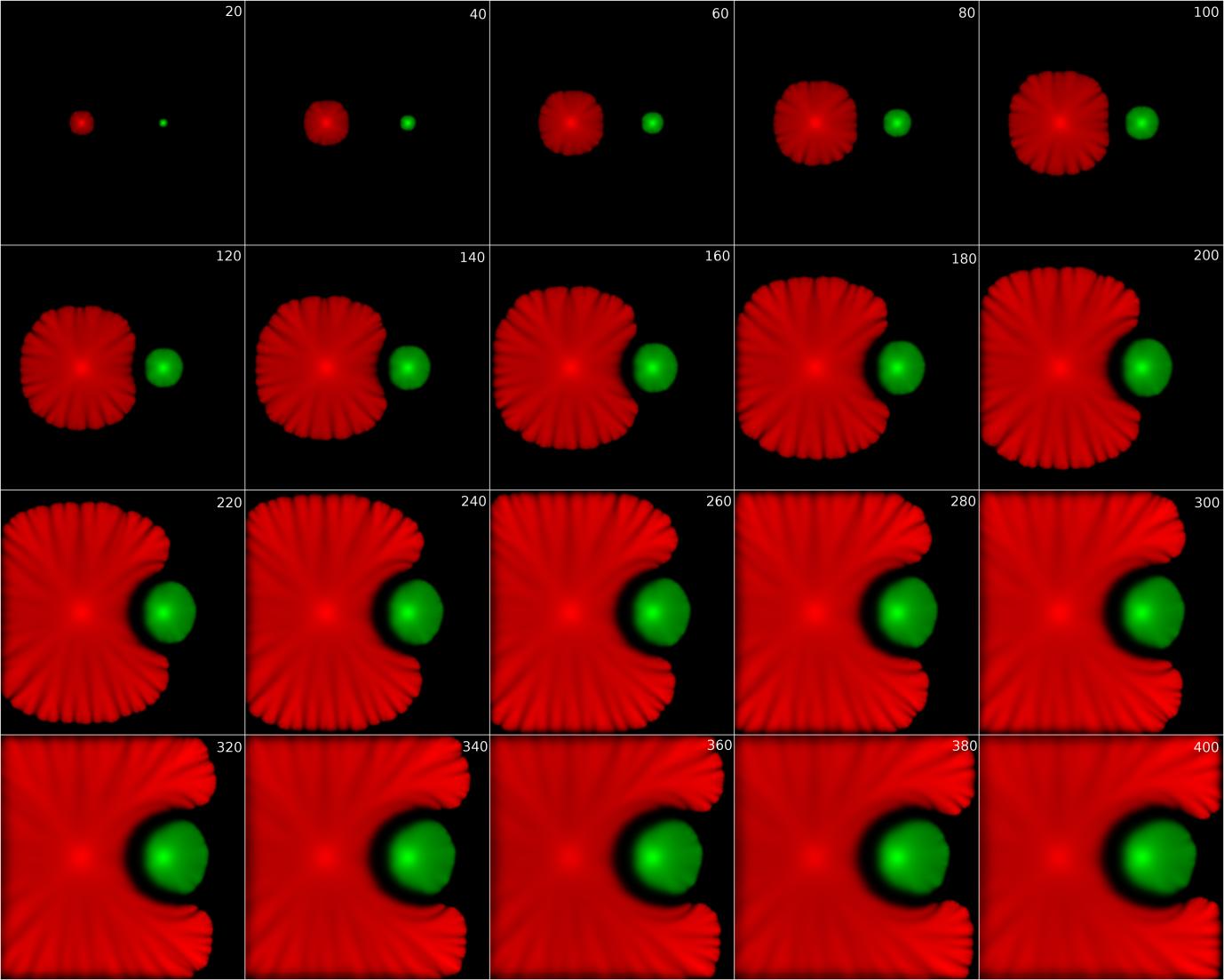


**Figure S3. Colony growth over time for random growth rates.** Colony growth dynamics when the growth rates a_1_ and a_2_ are taken to be random; a_1_= 0.8 + 0.4x; a_2_= 0.2 + 0.1x where x is a uniformly distributed random number lying between 0 and 1. Parameters used**:** $h_{c2}=0.5$, $\Gamma_{1}=0.01$; $\Gamma_{2}=0.005$; $\gamma_{2}=0.39$; $\lambda_{2}=1$; $\delta_{2}=0.01$; $d_{1}=0.001$; $d_{2}=0.0001$ ;$d_{h2}=0$; $c_{12}=0.4$; $\eta_{1}=0.4$


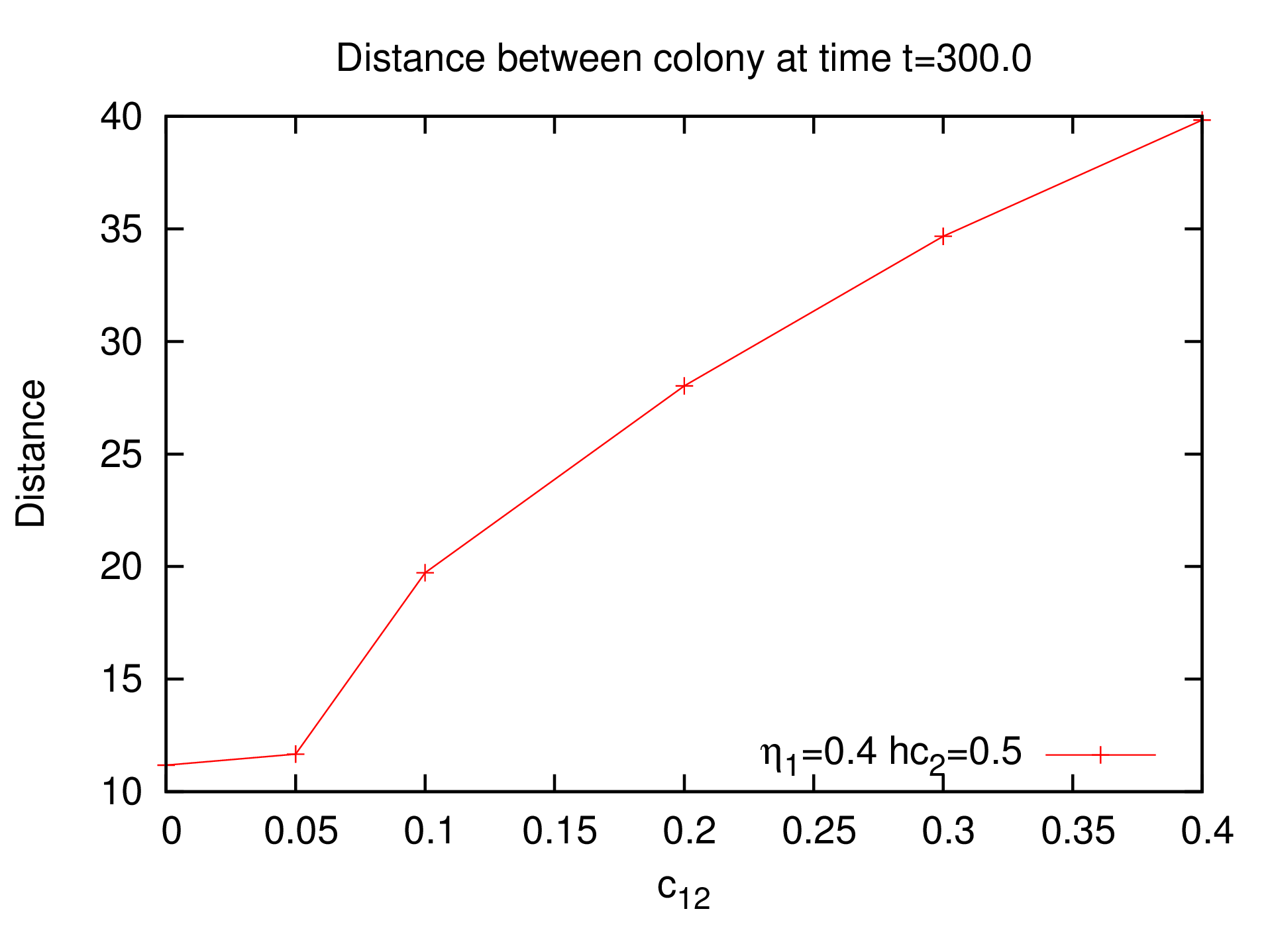


**Figure S4. Variation of minimum distance with chemotactic coefficient.** Variation of minimum distance between the two colonies with chemotactic coefficient. All other parameters are same as in Figure 6.


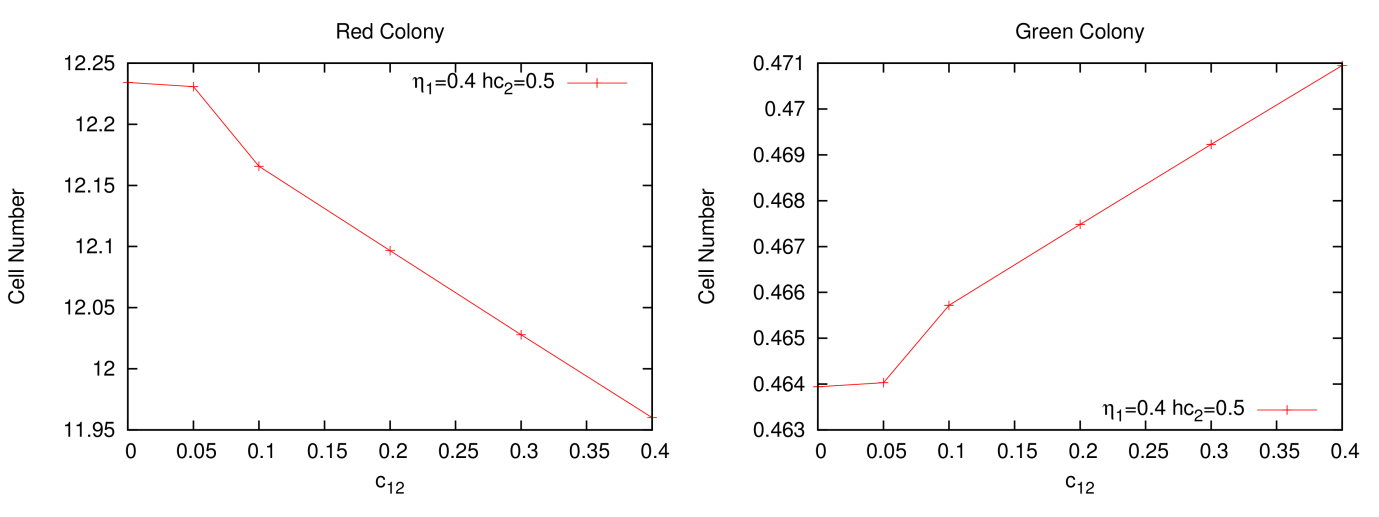
**Figure S5. Variation of cell population with chemotactic coefficient.** Variation of cell number with chemotactic coefficient. All other parameters are same as in Figure 6.

**References:**

1. [Ghoshal, S](http://www.ncbi.nlm.nih.gov/pubmed?term=Ghoshal S%5BAuthor%5D&cauthor=true&cauthor_uid=21872987)., [Bhattacharya, P](http://www.ncbi.nlm.nih.gov/pubmed?term=Bhattacharya P%5BAuthor%5D&cauthor=true&cauthor_uid=21872987). & [Chowdhury, R](http://www.ncbi.nlm.nih.gov/pubmed?term=Chowdhury R%5BAuthor%5D&cauthor=true&cauthor_uid=21872987). De-mercurization of wastewater by *Bacillus cereus* (JUBT1): growth kinetics, biofilm reactor study and field emission scanning electron microscopic analysis. *J. Hazard. Mater.* **194,** 355-361 (2011).

2. Xie, F., Zhang, Y., Li G, Zhou, L., Liu, S. & Wang, C. The CIpP Protease Is Requires for the Stress Tolerance and Biofilm Formation in Actinobacillus pleuropneumoniae. *PLOS ONE*. **8,** e53600. doi:10.1371/journal.pone.0053600 (2013).
